# A Multi-omics Approach to Unraveling the Microbiome-Mediated Effects of Arabinoxylan Oligosaccharides in Overweight Humans

**DOI:** 10.1128/mSystems.00209-19

**Published:** 2019-05-28

**Authors:** Alfonso Benítez-Páez, Louise Kjølbæk, Eva M. Gómez del Pulgar, Lena K. Brahe, Arne Astrup, Silke Matysik, Hans-Frieder Schött, Sabrina Krautbauer, Gerhard Liebisch, Joanna Boberska, Sandrine Claus, Simone Rampelli, Patrizia Brigidi, Lesli H. Larsen, Yolanda Sanz

**Affiliations:** a Microbial Ecology, Nutrition and Health Research Unit, Institute of Agrochemistry and Food Technology, Spanish National Research Council (IATA-CSIC), Paterna-Valencia, Spain; b Department of Nutrition, Exercise and Sports, Faculty of Science, University of Copenhagen, Frederiksberg C, Denmark; c Institute of Clinical Chemistry and Laboratory Medicine, University of Regensburg, Regensburg, Germany; d Leibniz Institute for Analytical Sciences (ISAS), Dortmund, Germany; e Department of Food and Nutritional Sciences, University of Reading, Reading, United Kingdom; f Microbial Ecology of Health Unit, Department of Pharmacy and Biotechnology, University of Bologna, Bologna, Italy; University of California, San Francisco

**Keywords:** AXOS, dietary fiber, glucose homeostasis, lipidomics, metabolic syndrome, metabolomics, microbiome, overweight

## Abstract

Long-term consumption of dietary fiber is generally considered beneficial for weight management and metabolic health, but the results of interventions vary greatly depending on the type of dietary fibers involved. This study provides a comprehensive evaluation of the effects of a specific dietary fiber consisting of a wheat-bran extract enriched in arabinoxylan-oligosaccharides (AXOS) in a human intervention trial. An integrated multi-omics analysis has been carried out to evaluate the effects of an intervention trial with an AXOS-enriched diet in overweight individuals with indices of metabolic syndrome. Microbiome analyses were performed by shotgun DNA sequencing in feces; in-depth metabolomics using nuclear magnetic resonance in fecal, urine, and plasma samples; and massive lipid profiling using mass spectrometry in fecal and serum/plasma samples. In addition to their bifidogenic effect, we observed that AXOS boost the proportion of *Prevotella* species. Metagenome analysis showed increases in the presence of bacterial genes involved in vitamin/cofactor production, glycan metabolism, and neurotransmitter biosynthesis as a result of AXOS intake. Furthermore, lipidomics analysis revealed reductions in plasma ceramide levels. Finally, we observed associations between *Prevotella* abundance and short-chain fatty acids (SCFAs) and succinate concentration in feces and identified a potential protective role of Eubacterium rectale against metabolic disease given that its abundance was positively associated with plasma phosphatidylcholine levels, thus hypothetically reducing bioavailability of choline for methylamine biosynthesis. The metagenomics, lipidomics, and metabolomics data integration indicates that sustained consumption of AXOS orchestrates a wide variety of changes in the gut microbiome and the host metabolism that collectively would impact on glucose homeostasis. (This study has been registered at ClinicalTrials.gov under identifier NCT02215343.)

**IMPORTANCE** The use of dietary fiber food supplementation as a strategy to reduce the burden of diet-related diseases is a matter of study given its cost-effectiveness and the positive results demonstrated in clinical trials. This multi-omics assessment, on different biological samples of overweight subjects with signs of metabolic syndrome, sheds light on the early and less evident effects of short-term AXOS intake on intestinal microbiota and host metabolism. We observed a deep influence of AXOS on gut microbiota beyond their recognized bifidogenic effect by boosting concomitantly a wide diversity of butyrate producers and Prevotella copri, a microbial species abundant in non-Westernized populations with traditional lifestyle and diets enriched in fresh unprocessed foods. A comprehensive evaluation of hundreds of metabolites unveiled new benefits of the AXOS intake, such as reducing the plasma ceramide levels. Globally, we observed that multiple effects of AXOS consumption seem to converge in reversing the glucose homeostasis impairment.

## Abstract (precis)

The use of dietary fiber food supplementation as a strategy to reduce the burden of diet-related diseases is a matter of study given its cost-effectiveness and the positive results demonstrated in clinical trials. This multi-omics assessment, on different biological samples of overweight subjects with signs of metabolic syndrome, sheds light on the early and less evident effects of short-term AXOS intake on intestinal microbiota and host metabolism. We observed a deep influence of AXOS on gut microbiota beyond their recognized bifidogenic effect by boosting concomitantly a wide diversity of butyrate producers and Prevotella copri, a microbial species abundant in non-Westernized populations with traditional lifestyle and diets enriched in fresh unprocessed foods. A comprehensive evaluation of hundreds of metabolites unveiled new benefits of the AXOS intake, such as reducing the plasma ceramide levels. Globally, we observed that multiple effects of AXOS consumption seem to converge in reversing the glucose homeostasis impairment.

## INTRODUCTION

The World Health Organization (WHO) reports that 1.9 billion adults are overweight, 650 million of whom are obese (1), making the obesity epidemic and the resulting metabolic complications an important health concern. The current rise in obesity prevalence in low- and middle-income countries, particularly in urban settings, and in children and adolescents further underlines the importance of prevention and treatment of obesity (1, 2). Obesity is the result of a long-term imbalance between energy intake and expenditure, mainly caused by overnutrition and sedentary lifestyle (3). Obesity is characterized by chronic low-grade inflammation and impairment of the lipid and glucose metabolism, thus increasing the risk of developing comorbidities such as type 2 diabetes (T2D), cardiovascular disease (CVD), and mental diseases (4, 5). Different epidemiological studies support the notion that regular consumption of enriched dietary fiber food is inversely correlated with weight gain, thus potentially reducing the risk of developing T2D and CVD (6–10). Interestingly, a recent umbrella review of systematic reviews reports that dietary fiber intake has a convincingly protective effect against CVD, including coronary artery disease and CVD-related death, and that there is suggestive evidence of disease risk reduction for several type of cancers, T2D, and stroke (11). Accordingly, dietary fiber consumption appears to be a feasible long-term and cost-effective strategy to prevent obesity and its comorbidities.

Dietary fiber comprises a diverse group of structurally complex carbohydrates with various effects on human metabolism and the gut microbiota (12). Arabinoxylans (AX) are cell wall components that constitute a major part of the dietary fiber fraction of cereal grains and are an important fiber source in the human diet (13). The main products of AX enzymatic hydrolysis are arabinoxylan-oligosaccharides (AXOS) and xylan-oligosaccharides (XOS), which show prebiotic potential because of their ability to increase the abundance of bifidobacteria and some butyrate producers in the gut (14–16). The bifidogenic effect of AX-derived oligosaccharides has been shown in human dietary interventions where abundance of *Bifidobacterium* species has been increased in feces as a consequence of the intake of 4-g/day XOS for 3 weeks (17) or 2- to 10-g/day AXOS during a similar period of time (18–20). Although no major impact on blood biochemical, physiological, and anthropometrical parameters has been observed following AXOS/XOS intake, most likely due to the short duration of the studies, the increases in fecal moisture (17) and short-chain fatty acid (SCFA) concentrations (19), and in postprandial ferulic acid concentration (20), altogether confirm utilization of this type of dietary fiber by gut microbiota and its potential impact on metabolic health.

**FIG 1:**
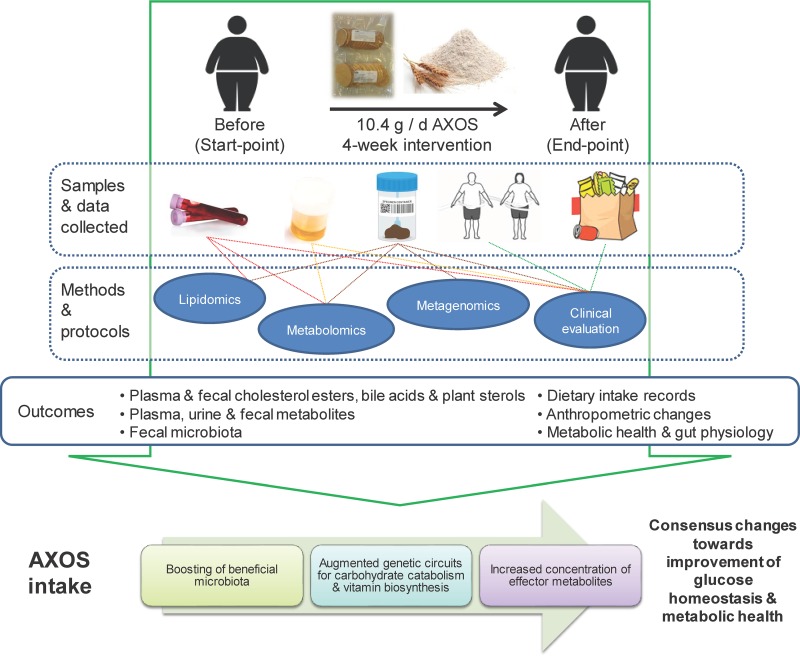
Graphical description of the study and the main outcomes assessed.

In a previous study including a randomized crossover intervention evaluating the effects of AXOS and polyunsaturated fatty acids (PUFA) in overweight subjects with indices of metabolic syndrome (MetS), we reported that AXOS exert a bifidogenic effect and increase the abundance of butyrate-producing bacteria using a 16S rRNA gene sequencing approach (21). In the current paper, we aimed to further characterize the microbiome, metabolome, and lipidome responses to AXOS intake to gain insight into the possible role of the microbiota as mediator of dietary effects on metabolic health (Fig. 1). To this end, we have analyzed the biological samples of the group of responders to AXOS consumption (who showed significant changes in their gut microbiota) from our previous study (Kjølbæk et al. [21]). A multi-omics approach has been applied, including shotgun DNA sequencing metagenomics of feces; nuclear magnetic resonance (NMR) metabolomics conducted in feces, plasma, and urine; and mass spectrometry-based (liquid chromatography mass spectrometry [LC-MS]) lipidomics in plasma and feces.

## RESULTS

### Human gut metagenome’s response to AXOS consumption. (i) Abundance changes in bacterial groups.

**FIG 2:**
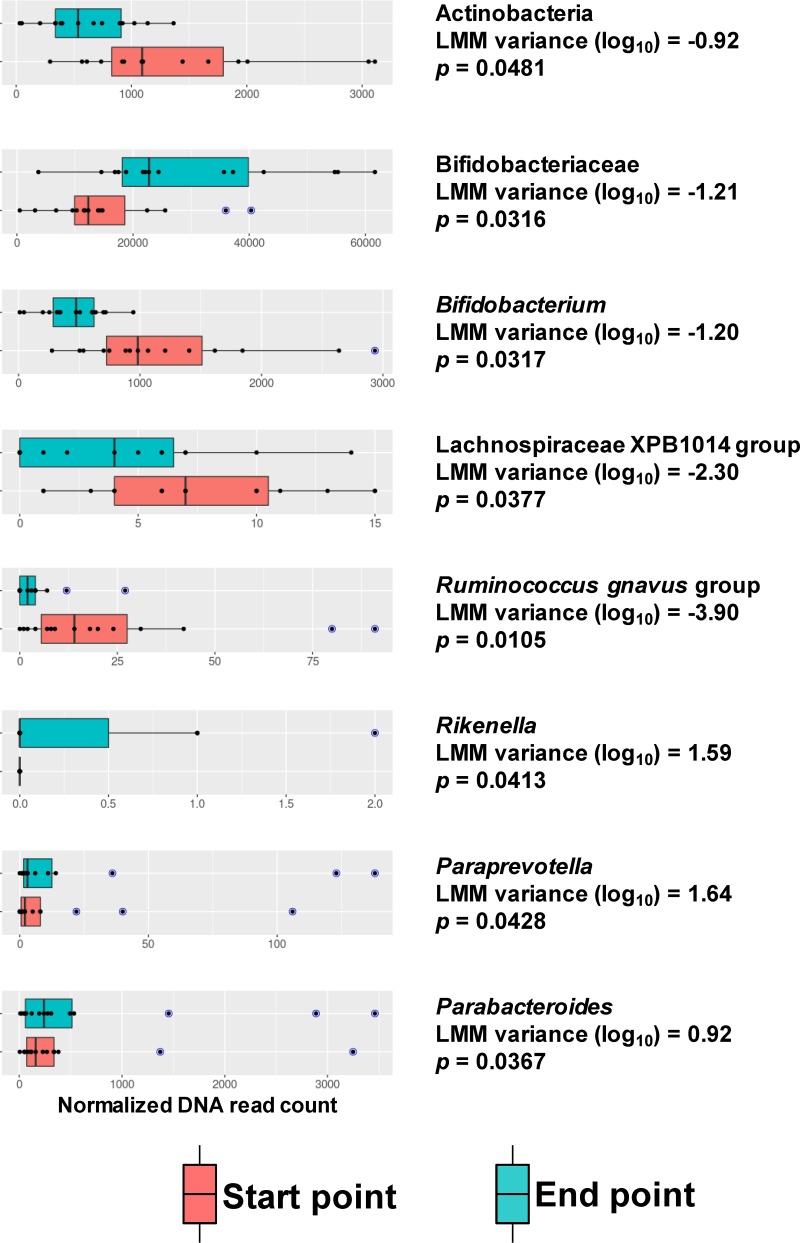
Gut microbiota components influenced by AXOS intake. The distribution of normalized reads belonging to the taxonomy categories with differential abundance after AXOS consumption is depicted in box plots. Red boxes represent start point samples (baseline before the intervention), whereas turquoise boxes represent the endpoint samples (at the end of the intervention). Blue data points indicate outliers. The linear mixed model (LMM) estimate (baseline as reference group) and *P* values are shown. LMM indicates the variance obtained using log-transformed data.

The new taxonomic analyses performed on metagenomic shotgun sequencing data indicate that AXOS intake increased the abundance of *Actinobacteria* (*P* ≤ 0.0481), *Bifidobacteriaceae* (*P* ≤ 0.0316), and *Bifidobacterium* (*P* < 0.0317) taxonomy groups, comparing samples at baseline with those after the intervention. In addition to this characteristic bifidogenic effect, the abundance of members of the Ruminococcus gnavus group (*P* ≤ 0.0105) and of the *Lachnospiraceae* XPB1014 group (*P* ≤ 0.0171) were also increased in samples after the AXOS intervention. Conversely, the AXOS intake reduced the proportion of *Rikenella* (*P* ≤ 0.0413), *Parabacteroides* (*P* ≤ 0.0367), and *Paraprevotella* (*P* ≤ 0.0428) species (Fig. 2).

**FIG 3:**
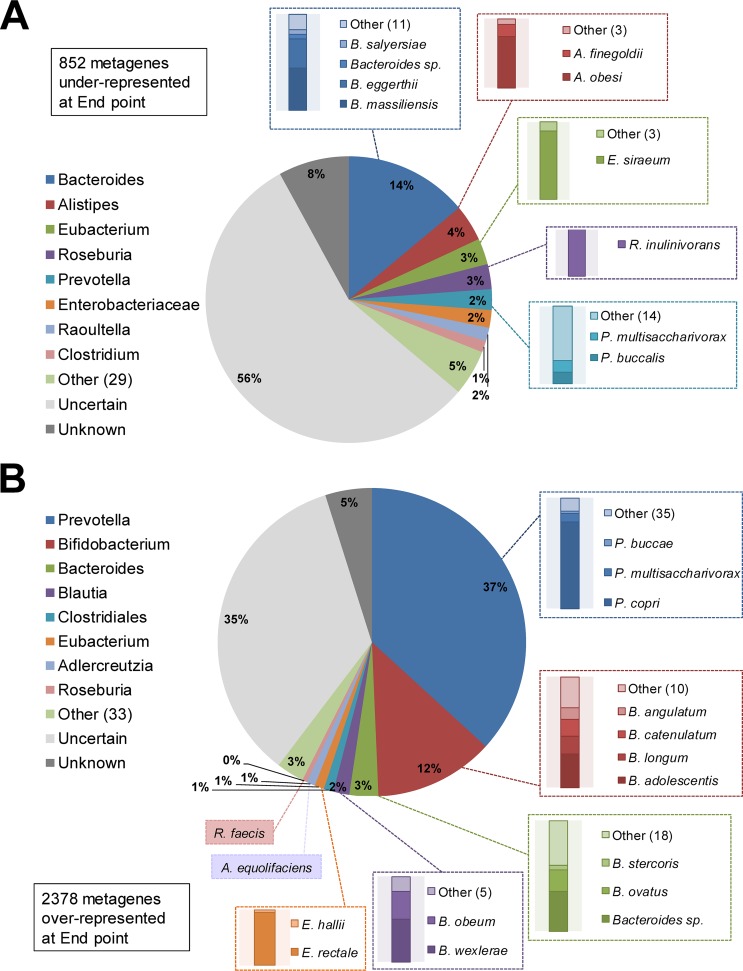
Taxonomy assessment of metagenes with differential abundance as a result of the AXOS intervention. (A) Genus and species distribution of the underrepresented metagenes in the metagenome of samples after AXOS intervention. (B) Similar analysis as in panel A for the overrepresented metagenes. Pie charts indicate the distribution at genus level, whereas bar plots indicate the distribution of the main species; the color coding is maintained accordingly. “Unknown” is used for taxonomic categories showing an identity score lower than 70% and no taxonomy defined at genus level. Those with a score identity higher than 70% and equal matching with several species from the different genera are shown as “Uncertain.” Numbers inside parentheses show the number of genera or species in addition to those shown in the graph.

The metagenome analysis was based on the processing of more than 3.2 billion reads (106 million paired-end reads on average per sample), which produced approximately 10.2 million encoding genes assembled. A nonredundant coding metagene database was obtained by clustering protein sequences predicted from sequencing reads, thus retrieving ∼1.7 million metagenes as a reference for the assessment of the effects of AXOS intake on the gut microbiome. We found that 3,230 metagenes (∼0.19% of the full data set) exhibited differential abundance, of which 852 were underrepresented and 2,378 were overrepresented after the intervention. In order to confidently disclose the taxonomy categories corresponding to those genes, we performed a simple BLASTP (https://blast.ncbi.nlm.nih.gov/Blast.cgi?PAGE=Proteins) search against the nonredundant protein database at the NCBI (release May 2017), making taxonomy assignments of query sequences with alignments of at least 70% amino acid sequence identity, compared with the respective top matches. Regarding the underrepresented set of metagenes, we were able to assign taxonomy categories for 36% of them with certainty and identify species of the genera *Bacteroides*, *Alistipes*, *Eubacterium*, *Roseburia*, *Prevotella*, and *Raoultella* (Fig. 3A). Of all the species, the metagenes of *Bacteroides* species were those most negatively influenced by AXOS intake, particularly Bacteroides massiliensis and Bacteroides eggerthii. Additionally, species such as Alistipes obesi, Eubacterium siraeum, Roseburia inulinivorans, Prevotella buccalis, and Prevotella multisaccharivorax were also reduced by AXOS intake. The analysis of the overrepresented set of metagenes (60% with reliable taxonomy identification) allow the identification of *Bifidobacterium* species, with Bifidobacterium adolescentis, Bifidobacterium longum, Bifidobacterium catenulatum, and Bifidobacterium angulatum as the most predominant (Fig. 3B). We observed that more than one-third of the overrepresented metagenes corresponded to *Prevotella* species, particularly Prevotella copri (Fig. 3B). We detected and quantified DNA reads for the six *Prevotella* phylotypes present in the SILVA database release 128 (see Fig. S1 in the supplemental material). Interestingly, each individual phylotype showed no significant distribution, including the “Prevotella_9” corresponding to *P. copri*, but with a notable increase in reads of “Prevotella_1” and “Prevotella_9.” Globally, the sum of reads for all *Prevotella* phylotypes indicated that such species are promoted by AXOS intake as well (*P* < 0.0501).

10.1128/mSystems.00209-19.1 FIG S1 Distribution of *Prevotella* phylotypes according to the SILVA database. The DNA read distribution for the six different *Prevotella* phylotypes found to be annotated in the SILVA database (release 128) is drawn in a box plot. The identity for each phylotype was deduced from the direct comparison of the database reads against the nonredundant 16S NCBI database, thus selecting the matches with top score and sequence identity to assign the taxonomy at the species level. Red boxes show the distribution in start point samples, whereas the turquoise boxes indicate the distribution in the endpoint samples. Blue data points indicate the outliers, and the Wilcox signed-rank estimates (W) and *P* values are shown below the box plots. Download FIG S1, TIF file, 2.9 MB. Copyright © 2019 Benítez-Páez et al. 2019 Benítez-Páez et al. This content is distributed under the terms of the Creative Commons Attribution 4.0 International license.

The analysis of the overrepresented set of metagenes also enabled the identification of additional bacterial species, such as Bacteroides stercoris, Bacteroides ovatus, Blautia obeum, Blautia wexlerae, Eubacterium rectale, Eubacterium hallii, Roseburia faecis, and Adlercreutzia equolifaciens, which were increased after AXOS intervention (Fig. 3B).

**FIG 4:**
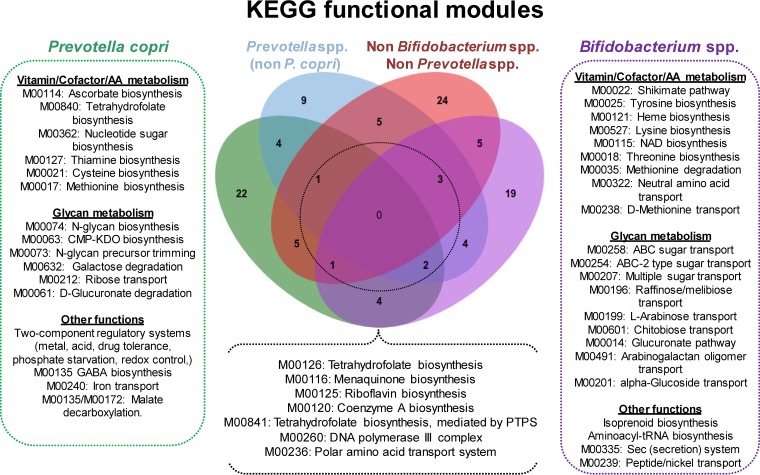
Gain of function in the gut microbiome as a consequence of AXOS ingestion. Overrepresented metagenes from *Bifidobacterium* spp. (*n* = 315), Prevotella copri (*n* = 782), non-*copri Prevotella* spp. (*n* = 415), and non-*Bifidobacterium*/non-*Prevotella* species (*n* = 866) were mapped to the KEGG database and then assigned to the respective functional modules of that repository. A Venn analysis displays the taxonomy-specific functions gained and those exacerbated by the simultaneous presence in at least 3 out of the 4 groups of bacterial species analyzed (dashed circle).

**(ii) Functional analysis.** The set of metagenes underrepresented as a consequence of AXOS intake exhibits a wide range of metabolic and cellular functions according to the annotation with KEGG. Of these, some metagenes encode potential pathogenic features, such as biofilm formation (ko:02026, ko:02025, and ko:05111), beta-lactam resistance (ko:01501), bacterial secretion systems for virulence factor release (ko:03070), flagellar assembly (ko:02040), cationic antimicrobial peptides (CAMP resistance) (ko:01503), bacterial chemotaxis (ko:02030), monobactam biosynthesis (ko:00261), and lipopolysaccharide biosynthesis (ko:00540) (Table S2). When we analyzed the overrepresented set of metagenes, we found a lower level of functional annotation than for the underrepresented set of metagenes (24% and 43%, respectively). We then evaluated the function of those metagenes present in the species predominantly influenced by AXOS, such as Prevotella copri and *Bifidobacterium* spp., which account for roughly 50% of the overrepresented metagenes (Fig. 3B). As shown in Fig. 4, the AXOS consumption led to an expansion of the collection of genes dedicated to vitamin and cofactor biosynthesis as well as of genes specialized in transport, biosynthesis, and degradation of glycans. Notably, we also found a significant increase of genes involved in the biosynthesis of the neurotransmitter gamma-aminobutyric acid (GABA) and precursors of aromatic amino acids (shikimate and chorismate) with potential roles in the gut-brain communication. Moreover, the Venn analysis indicated that the functions amplified to a greater extent by AXOS intake (present in at least 3 out of the 4 groups of overrepresented metagenes) consisted of those involved in the biosynthesis of vitamin K_2_, the derivative vitamin B_9_ tetrahydrofolate (THF), vitamin B_2_, and the coenzyme A biosynthesis. All the above were equally found to be overrepresented in metagenes from Prevotella copri and *Bifidobacterium* species (Fig. 4).

To investigate the bacterial cellular functions boosted by AXOS consumption, we conducted a domain enrichment analysis using the Pfam annotation system. This showed that the overrepresented metagenes were almost exclusively dedicated to glycan metabolism, with several metagenes exhibiting a wide range of particular functions for this fundamental cell process (Table S3). We observed overrepresentation of functions such as outer membrane proteins (OMPs) for carbohydrate binding and transport relying on OMP-, TonB-, and SusD-like proteins. Moreover, we also identified a vast amount of proteins enriched in carbohydrate binding modules (CBMs) characteristic of glycosyl hydrolase enzymes such as beta-galactosidases, of which central domains were likewise enriched in the overrepresented metagenes. More-general functions were also found in several metagenes that encode ATPases with no defined molecular roles but apparently related to sugar transport function according to associations inferred from functional and phylogenetic information. Increases in the presence of metagenes encoding carboxypeptidases and protein domains associated with similar functions (PD40/WD40-like) suggested that AXOS could increase the activity of specific peptidases of the gut metagenome. An increased presence of several metagenes associated with the prokaryote innate immune response, such as restriction modification systems, was also observed (Table S3).

**(iii) Human gut metavirome response to AXOS intake.** We also used the metagenome information to evaluate the presence of virus sequences in the raw data retrieved from shotgun sequencing of fecal DNA. By using the ViromeScan approach (22), we were able to evaluate the relative abundance of approximately 22 virus families. In general terms, we found a predominant tendency toward a decrease of reads matching human virus sequences after the AXOS intervention, although the differences did not reach statistical significance. Interestingly, our viral characterization on the gut samples led to the detection of *Megavirales* and other giant viruses (>200 nm in diameter), which were only recently identified in human stool and other human samples (23). The clinical or biological significance of the presence of these viruses in the human gut remains to be determined; for this reason, our data pave the way to searching for giant viruses in the human gut and to establishing the impact that diet has on them.

**AXOS impact on markers of glucose homeostasis and gut microbiota.** A new evaluation in the set of responders indicates that AXOS intake did not result in significant changes in most of the metabolic health outcomes and biomarkers analyzed (anthropometry, inflammatory, lipid, or glucose biomarkers), probably due to the limited sample size and short study duration. Nevertheless, we observed that the mean value of fasting insulin as well as of the insulin resistance index (homeostatic model assessment for insulin resistance [HOMA-IR]) was slightly reduced (Fig. S2A, B, and C). Although this reduction was observed in only a proportion of the subjects analyzed (∼47%), it is suggestive of a potential improvement in glucose metabolism probably linked to changes in certain microbiota signatures. Using a logistic regression analysis, we found that changes in the HOMA-IR tended to be related to three different taxonomy groups, whose abundance was increased as a result of the intervention (Fig. S2D). In particular, the changes in abundance of “*Lachnospiraceae* AC2044 group” (odds ratio = 4.95, confidence interval [CI] = 4.39 to 49.65, *P* = 0.0571), “Eubacterium ruminantium group” (odds ratio = 3.53, CI = 1.22 to 20.15, *P* = 0.0575), and “*Lachnospiraceae* XPB1014 group” (odds ratio = 3.20, CI = 1.05 to 15.69, *P* = 0.0737) suggest that an increase of 1 logarithmic unit in the relative abundance of these microbial groups leads to an increase of at least 3-fold in the probability of the HOMA-IR amelioration.

10.1128/mSystems.00209-19.2 FIG S2 Glucose metabolism and relationship with gut microbiome. The fasting plasma glucose and insulin concentrations are depicted in box plot manner for samples before (Start point) and after (End point) the intervention (A and B, respectively). Similarly, the HOMA-IR values derived from the integration of the above biochemical parameters are shown (C). A logistic regression suggesting the gut microbiota groups potentially related to improvements in the HOMA-IR, based on their abundance changes across the intervention, is shown in panel D. The *P* values are shown on top of the respective box plots. Download FIG S2, TIF file, 1.1 MB. Copyright © 2019 Benítez-Páez et al. 2019 Benítez-Páez et al. This content is distributed under the terms of the Creative Commons Attribution 4.0 International license.

### AXOS effects on plasma and fecal lipidome.

By using MS-based lipidomics approaches, we were able to quantify 9 lipid classes and more than 150 species according to the chain length and double bond content in both plasma and fecal samples. We found no significant changes in the lipid profiles from feces in response to the intervention. In plasma, we detected a significant increase in one out of 18 analyzed cholesteryl ester species, CE 20:0, and a decrease in two hexosylceramides and one ceramide (Table S4). For the latter, a quasiconsensus signal of reduction in plasma ceramide levels was observed in 8 out of the 9 features evaluated (three of them in a significant manner, *P* < 0.013), thus obtaining a global trend for a decrease in the total ceramide content in plasma samples (*P* ≤ 0.0788). We found no correlations between microbiome signatures and this lipidomic feature.

### Metabolomic profiling upon AXOS consumption.

**FIG 5:**
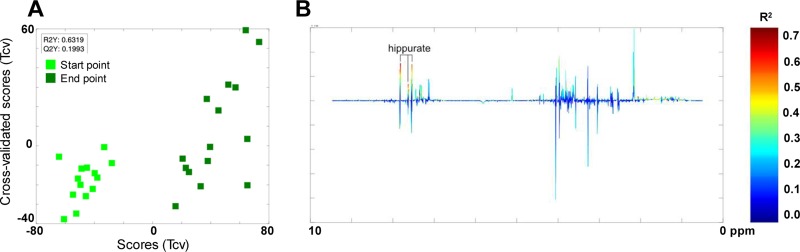
Effects of the dietary intervention on urine metabolome. The urinary metabolome was analyzed by ^1^H nuclear magnetic resonance. Orthogonal projection to latent structure discriminant analysis models were used to compare the changes in the urinary metabolome at start points and endpoints. (A) For this pairwise comparison, the plot of the scores (T) compared with cross-validated scores (Tcv) is shown. (B) Loading plot is color coded according to the correlation coefficient (*R*^2^) with *Y* (predictor vector coding time of the intervention). Q2, goodness of prediction; R2, goodness of fit.

The NMR-based metabolomic analysis of the metabolite profile before and after the AXOS intervention indicated that there were changes only in the urine hippuric acid concentration (Fig. 5). This increase was more prominent in a subgroup of participants, which could be attributed to higher abundance of *Prevotella_6*-associated species (*R*^2^*Y* = 0.5951, *Q*^2^*Y* = 0.1320) and negatively correlated with *Ruminococcaceae UCG.012* abundance (*R*^2^*Y* = 0.7190, *Q*^2^*Y* = 0.2795). Additional correlation patterns suggested that urine dimethylamine (DMA) levels were associated with proportions of *Peptoclostridium* species (*R*^2^*Y* = 0.6010, *Q*^2^*Y* = 0.2679). In fecal samples, positive correlations between *Prevotella_2* abundance and the concentrations of acetate, propionate, and succinate were identified (*R*^2^*Y* = 0.7357, *Q*^2^*Y* = 0.1818). Finally, application of a supervised model of analysis allowed partial discrimination between plasma samples before and after the intervention (start and end points). Signals from residual very-low-density lipoprotein (VLDL) and triglyceride (TAG) fractions, including fatty acids and glyceryls of lipids, were to some extent increased after the intervention. Although this could be indicative of a trend for dyslipidemia, the VLDL profile in plasma measured by classical biochemical methods indicates no major changes in this type of lipid during the intervention (*P* ≤ 0.2099). On the other hand, the abundance of Eubacterium rectale, a microbial species boosted by AXOS intake (Fig. 3B) (21), was positively correlated with concentrations of phosphocholine and *sn*-glycero-3-phosphocholine in plasma (*R*^2^*Y* = 0.5468, *Q*^2^*Y* = 0.2928).

## DISCUSSION

Using a multi-omics approach, we have performed an in-depth characterization of the microbiome, lipidome, and metabolome response to a specific source of fiber (AXOS). In previous intervention trials, there has been controversy on the effect of AXOS on health outcomes with the microbiome assessments being limited to taxonomic features (18, 19, 21, 24, 25). The present study aimed at providing a more exhaustive analysis of the AXOS-driven metabolic changes with a particular focus on the early gut microbiota-induced changes after a 4-week randomized dietary intervention. In agreement with previous findings, we found that a bifidogenic effect of AXOS was evident when the metagenome was analyzed using shotgun sequencing (18–20, 26). In this study, we report new microbiome effects resulting from AXOS intake, including increases in the abundance of Ruminococcus gnavus and *Lachnospiraceae* XPB1014 taxonomy groups, the latter related to *Eubacterium*, *Blautia*, and *Roseburia* species (BLASTN search against the nonredundant 16S NCBI database, 91 to 94% sequence identity). We have described the AXOS impact on Blautia luti *and* Blautia wexlerae species and other butyrate/propionate-producing bacteria such as Eubacterium rectale, Eubacterium hallii, Faecalibacterium prausnitzii, and Dorea longicatena, based on V3-V4 16S amplicon sequencing data and the combination of multivariate and linear analyses of operational taxonomic units (OTUs) in a previous paper (21). Moreover, we observed that AXOS intake significantly reduced *Parabacteroides*, *Paraprevotella*, and *Rikenella* species. Similarly, reductions in *Parabacteroides* proportions have been linked to improvement of glucose homeostasis in obese mice after dietary supplementation with capsaicin according to another study (27). This effect produced by AXOS on *Paraprevotella* species was also observed after the Roux-En-Y gastric bypass (RYGB)-like and metformin treatments in the Zucker diabetic fatty (ZDF) rat model of obesity and T2D (28).

Upon assembling and predicting the set of bacterial genes present in the gut microbiome of responders included in the present study, we also found that AXOS intake increased the proportion of *Prevotella* species and particularly of Prevotella copri. When searching the SILVA database (release 128), we found that of the six different phylotypes present in the database, only two tend to be increased after AXOS intake (Prevotella_1 and Prevotella_9). However, a change in this taxonomy category (*P* < 0.0501) was detected as a result of the intervention when all *Prevotella* phylotypes were combined and considered in the analysis. These findings could lead to questioning of the proper identification of this complex group of bacteria. This issue has been reported previously in a human study where De Filippis and coworkers describe that correlation patterns of *Bacteroides* and *Prevotella* species with animal- or plant-derived nutrients, respectively, correspond with a simplistic association given the unexplored genetic variability at the subgenus level of these taxonomic groups prevalent in the human gut microbiota (29).

The role of the species *P. copri* in the amelioration of glucose intolerance seems to be contradictory according to the existing scientific evidence. While the early metagenomic assessment of the gut microbiome of T2D patients indicated that *P. copri* is linked to an increased production of branched-chain amino acids (in plasma and gut) associated with insulin resistance and to a higher risk of T2D (30), other animal studies reported that *P. copri* has a positive impact on glucose homeostasis, given the activation of the intestinal gluconeogenesis via succinate production (31–33). Therefore, it is difficult to attribute a healthy or harmful role to *P. copri* in glucose homeostasis in the light of the existing evidence. We hypothesize that such antagonist effects could depend on the specific strains tested, on the substrate availability (activating different proteolytic or saccharolytic pathways), and on other components of the gut microbiota involved in host-microbe and microbe-microbe interactions and on the experimental conditions. In fact, a very recent work of De Filippis et al. suggests that antagonist associations of *P. copri* strains with health and disease would be explained by genetic variability and certain metabolic traits (34). In our particular case, AXOS intake increased a large number of metagenes carried by *P. copri* and tended to improve the HOMA-IR index (*P* = 0.0837), suggesting that AXOS intake can ameliorate the glucose metabolism, via modification of metagenomic functions, in overweight individuals with signs of MetS, in agreement with other studies in humans (25, 35). Nevertheless, this slight improvement in HOMA-IR values was not explained by a change in abundance of any *Prevotella* phylotype, including those closely related to *P. copri*. In contrast, we unveiled an association trend between the HOMA-IR and three different taxonomy groups, including the *Lachnospiraceae* XPB1014 group related to *Eubacterium*, *Blautia*, and *Roseburia* species. Special attention should be given to the species from the *Lachnospiraceae* XPB1014 group since these were found to be augmented by AXOS intake (Fig. 2). It is likely that several components of the microbiota involved in the generation of SCFAs by cross-feeding mechanisms could explain potential AXOS effects on glucose metabolism. Such effects could be mediated, for example, by the conversion of succinate into SCFAs like propionate, which can trigger intestinal gluconeogenesis and improve glucose metabolism (32).

From a functional point of view, we observed that AXOS consumption increased the abundance of genes involved in carbohydrate metabolism as well as vitamin and cofactor biosynthesis. The expansion of the set of glycosyl hydrolases and enzymes related to carbohydrate metabolism, essentially in *P. copri* and *Bifidobacterium* species, could be involved in the generation of organic acids, including SCFAs, through cross-feeding mechanisms with potential beneficial effects on gut and metabolic health (16). Theoretically, microbiome-mediated increases in the production of vitamins and cofactors could also bring host benefits (e.g., vitamin K_2_ in bone physiology altered in obese individuals [36, 37]), although direct evidence of vitamin production and host utilization remains to be shown. The AXOS intake increased the representation of metagenes involved in the biosynthesis of tetrahydrofolate (THF; the active form of folic acid), which may also have potential metabolic benefits. Indeed, some obese individuals show folate deficiency, which could lead to hyperhomocysteinemia related to atherosclerosis risk (38). In line with this, administration of folic acid and derivatives seems to confer protection against hyperhomocysteinemia-induced oxidative stress (39). AXOS intervention also increased the capacity of the gut microbiome for aromatic amino acid production (shikimate pathway producing precursors for tryptophan, phenylalanine, and tyrosine biosynthesis), which theoretically could increase the generation of derivative neurotransmitters (e.g., serotonin, dopamine, epinephrine, and norepinephrine). An augmented capacity for GABA biosynthesis was also detected as a consequence of AXOS intake. Altogether, this could result in additional effects on the communication within the gut-brain axis (40). Different studies based on mouse models indicate that low concentrations of GABA are associated with anxiety-like behavior, insulin resistance, energy expenditure imbalance, and weight gain in overweight and obesity (41–44). Particularly, one animal study indicated that GABA acts as activator of insulin secretion, given that GABA-like immunoreactive cells in the pancreas of rats are reduced in diabetes (45). A recent study in obese rats also indicated that GABA supplementation reduces serum ceramide concentrations (43). Therefore, we hypothesized that the AXOS-driven increased abundance of microbial species encoding GABA production could hold promise for the mediation of metabolic and mental health effects. Interestingly, other microorganisms like *Bacteroides* species, evolutionarily related to the *Prevotella* genus members, as well as some *Lactobacillus* and *Bifidobacterium* species, also have been described to be GABA-producing gut microbes (46, 47). Our findings also show that AXOS intake significantly decreases the concentration of several ceramide species in plasma (*P* < 0.013). Previous reports have also shown an association between the increased levels of ceramides and impaired glucose homeostasis in MetS and T2D patients (48–50).

Finally, the metabolomics approach was useful to trace the utilization of AXOS by gut microbiota through the presence in urine of hippuric acid, a microbial metabolite derived from degradation of polyphenols associated with dietary fiber (51). Our data suggest that hippurate production could be related to the abundance of *Prevotella* species increased by AXOS intake. Additionally, acetate, propionate, and succinate concentrations in feces were also positively related to the abundance of *Prevotella* species, which could bring benefits to metabolic health as explained above (32). The plasma metabolomic profiling indicates that phosphatidylcholine biosynthesis could be increased by AXOS intake via the butyrate producer Eubacterium rectale, which showed positive correlations with phosphatidylcholine precursors (*P* < 0.05). In turn, this could decrease bioavailability of choline for the production of methylamines (DMA and trimethylamine [TMA]) by other gut microbes (52), such as *Peptoclostridium* species that showed a positive correlation with the DMA urine concentration in our study. TMA is the precursor of trimethylamine oxide (TMAO), which is produced in the liver, kidney, and other tissues. Increased plasma levels of TMAO have been associated with enhanced risk of developing atherosclerosis, T2D, and chronic kidney disease. Therefore, this could be a mechanism by which AXOS microbiome-mediated effects could confer protection against the impairment of glucose metabolism, (53), as well as against a wide variety of chronic diseases (54), reducing the availability and exposure to methylamine oxide forms. Indeed, a previous study in patients with chronic kidney diseases found that AXOS slightly decreased plasma TMAO (55), results that further support our ideas.

### Conclusions.

Using a multi-omics functional approach, we have characterized in depth the effects of AXOS intake on the microbiome, lipidome, and metabolome and, thereby, tentatively identified possible microbiome- and non-microbiome-mediated dietary effects on metabolic health. We have shown that in addition to the well-recognized bifidogenic effect of AXOS, this type of dietary fiber increases the abundance of *Prevotella* and clostridial bacteria from the *Lachnospiraceae* family (butyrate producers) along with increases in organic acids (propionate and succinate). Furthermore, AXOS decreases plasma ceramide levels via a microbiome-independent mechanism, which altogether could contribute to improved glucose metabolism. The direction of other functional metagenomic changes induced by AXOS was related to the production of neuroactive (GABA) and choline metabolites, suggesting potential additional effects that could reduce the risk of developing chronic metabolic conditions. Studies with a larger sample size and longer duration are warranted to confirm whether the metabolome and lipidome profiles resulting from AXOS intake and the induced microbiome configuration are translated into metabolic health outcomes, aiding in the validation of the biomarkers of dietary exposure and function tentatively identified in the present study.

## MATERIALS AND METHODS

### Ethics approval and consent to participate.

The study is registered at ClinicalTrials.gov (NCT02215343), was conducted according to the guidelines laid down in the Declaration of Helsinki, and was carried out in accordance with the ethical standards of the responsible regional committee on human experimentation in Denmark, registered as H-4-2014-052, and the Danish Data Protection Agency (2013-54-0522).

### Study design.

The present study is based on a non-placebo-controlled randomized crossover trial with two dietary intervention periods (4 weeks each) separated by a washout period (4 weeks) conducted in 30 overweight and obese individuals (body mass index [BMI] of 25 to 40 kg/m^2^) with MetS index (an increased waist circumference plus at least one of the criteria for MetS [56]) described in detail elsewhere (21). The dietary fiber ingredient provided was a wheat bran extract, enriched in arabinoxylan-oligosaccharides (AXOS) (10.4 g/day AXOS). Before and after intervention periods, weighted dietary records and fecal, urine, and blood samples as well as anthropometric measurements were collected (Fig. 1). The present study encompasses the integration of clinical and multi-omics data derived from multiple biological samples of a subset of responders to AXOS intervention (*n* = 15) (Fig. 1), for whom AXOS did produce meaningful shifts in the structure of the gut microbial community (with no risk of carryover effect because of the crossover design). The information inferred from beta-diversity analysis, based on the Bray-Curtis dissimilarity index among samples (PERMANOVA = 1.90, *P* < 0.02), was used as an indicator of significant changes in the microbial community structure (21). The baseline characteristics of this cohort of samples are described in Table S1 in the supplemental material. Responders were defined as the group of participants included in the same dietary intervention period and showing significant changes in their microbiota composition, for whom the OTUs matching with bifidobacteria and/or recognized butyrate producer species were boosted as a result of the dietary intervention with AXOS and assessed by16S rRNA gene sequencing (21).

10.1128/mSystems.00209-19.3 TABLE S1 Characteristics for the participants involved in the multi-omics assessment. (1) Data are given for baseline (Start point) as median with Q1; Q3 distribution. (2) *n* = 14; enough blood sample from one participant could not be obtained for biochemical analysis for technical reasons. ALAT, alanine aminotransferase; ASAT, aspartate aminotransferase; BMI, body mass index; CHO, cholesterol; FM, fat mass; Hb, hemoglobin; HDL, high-density lipoprotein; HOMA-β, homeostatic model assessment, beta-cell function; HOMA-IR, homeostatic model assessment, insulin resistance; hsCRP, highly sensitive C-reactive protein; LBM, lean body mass; LDL, low-density lipoprotein; TG, triglycerides; VLDL, very-low-density lipoprotein; WBC, white blood cell count. Download Table S1, DOCX file, 0.01 MB. Copyright © 2019 Benítez-Páez et al. 2019 Benítez-Páez et al. This content is distributed under the terms of the Creative Commons Attribution 4.0 International license.

10.1128/mSystems.00209-19.4 TABLE S2 KEGG metabolic pathways associated with the down-represented metagenes. Download Table S2, DOCX file, 0.01 MB. Copyright © 2019 Benítez-Páez et al. 2019 Benítez-Páez et al. This content is distributed under the terms of the Creative Commons Attribution 4.0 International license.

10.1128/mSystems.00209-19.5 TABLE S3 Functional enrichment analysis in the overrepresented metagenomes. (1) Domain identifier and functional annotation according to information retrieved from the Pfam database (http://pfam.xfam.org/). (2) Odds ratio (OR) calculated from Fisher’s exact test under conditional maximum likelihood estimate (MLE) approach. (3) False-discovery rate (FDR) obtained after multiple testing correction of all domains detected (*n* = 7,711 domains). Download Table S3, DOCX file, 0.02 MB. Copyright © 2019 Benítez-Páez et al. 2019 Benítez-Páez et al. This content is distributed under the terms of the Creative Commons Attribution 4.0 International license.

10.1128/mSystems.00209-19.6 TABLE S4 Changes in lipid profiles from plasma samples. (1) *P* values are based on comparison using Wilcox signed-rank test and corrected by multiple testing using the Benjamini-Hochberg method. Cer, Ceramide; HexCer, hexosylceramide; CE, cholesteryl ester. Download Table S4, DOCX file, 0.01 MB. Copyright © 2019 Benítez-Páez et al. 2019 Benítez-Páez et al. This content is distributed under the terms of the Creative Commons Attribution 4.0 International license.

### Sampling. (i) Urine and feces.

Collection of fecal and urine samples took place at home prior to the clinical investigation day (CID) in sterile collection tubes provided. The urine sample was collected in the morning, and the fecal sample was collected as close to the urine sample as possible. Both samples were kept at 4°C after collection and delivered to the Department of Nutrition, Exercise, and Sports, University of Copenhagen, within 3 h after collection of feces. At the Department, the samples were weighed and the density of the urine sample was measured (Atago PAL-10S pocket refractometer) to calculate the volume. For the metabolomic analysis of urine, an aliquot of 4 ml urine was used and 45 μl 0.1% sodium azide was added. For fecal analysis, an aliquot of the sample was transferred to the EasySampler kit for stool collection (GP Medical Devices, Denmark) for metagenomic analyses. Here, the fecal sample was homogenized with MilliQ water, 1:1, and an aliquot was used for metabolomic analyses. For lipidomic analysis, 500 μl methanol was added per 500 mg homogenized fecal sample. All aliquots were stored at −80°C.

**(ii) Blood samples.** Prior to the CID, the participants consumed a standardized dinner in the evening followed by a fasting period of at least 8 h. All blood samples were collected in the fasting state. Lipidomic analyses were conducted in plasma and serum. For plasma analysis, blood was collected in EDTA tubes and put directly on ice, and for serum analysis, blood was kept at room temperature for 20 min to coagulate before centrifugation. For metabolomic analysis, blood for plasma analyses was collected in heparin tubes and put directly on ice. Afterwards, all blood samples were centrifuged at 2,500 × g for 10 min at 4°C to obtain respective aliquots of serum and plasma and stored at −80°C. Sampling procedures for plasma and serum samples presented here—for example, glucose, insulin, lipids, inflammation markers, etc.—are found elsewhere (21).

### Metagenomics approach. (i) DNA extraction and shotgun sequencing.

The fecal DNA was extracted using the QIAamp Fast DNA stool minikit (Qiagen, Hilden, Germany) according to the manufacturer’s instructions with a prior step of bead beating in 2-ml microcentrifuge tubes containing 0.1-mm-diameter glass beads, ∼200 mg feces, and 1 ml InhibitEX buffer. Bead beating was carried out in a Mini-Bead Beater apparatus (BioSpec Products, Bartlesville, OK, USA) with two cycles of shaking during 1 min and incubation on ice between cycles. The fecal DNA was quantified through Qubit 3.0 and the Qubit dsDNA HS assay kit (Thermo Fisher Scientific, Waltham, MA, USA), and 1.5 μg DNA of every sample was sent to be multiplexed and sequenced in a plate of the HiSeq2500 platform with 2 × 125 paired-end configuration (Eurofins Genomics GmbH, Ebersberg, Germany). The NEBNext Ultra DNA library prep kit for Illumina was used according to the manufacturer’s instructions with 400- to 500-bp insert size and low PCR cycling (5 cycles).

**(ii) Metagenome data analysis.** Approximately 0.5 Tb raw data were delivered in fastq files. Paired-end fastq files were used to assemble the fecal metagenome of each individual at two different time points by using Velvet assembler v 1.2.10 (57) with k-mer length 61, *-exp_cov* auto, and *-ins_length* 200 parameters, followed by an assembly refinement step using the Metavelvet extension (58) with the *-ins_length* 200 *-ins_length_sd* 50 configuration. The assembled contigs larger than 200 nucleotides (nt) in length were retained, and the prediction of potential open reading frames (ORFs) contained in such fragments from respective metagenomes was assisted by FragGeneScan v1.30 (59), with the *-complete*=0 and *-train*=complete configuration. Peptide sequences obtained from the ORF prediction in all metagenomes were concatenated and clustered at 70% sequence identity using *cdhit* algorithm with *-c* 0.7, *-G* 1, *-M* 10,000, *-B* 1, and *-g* 1 parameters (60, 61). For read mapping against the nonredundant peptide database compiled from the 30 metagenomes assembled, we used the *Usearch* v8.0.1623 algorithm with the following parameters: *-usearch_local*, *-id* 0.7, *-strand* both, and *-maxaccepts* 1. Differential abundance of coding metagenes was assessed by using negative binomial distribution methods implemented in edgeR (62) and determining a false-discovery rate (FDR) for selection of <0.1. For taxonomy aims, we mapped the remaining set of reads with no hits after comparison against the nonredundant coding database. Consequently, we mapped those reads against the reference Silva database (release 128, https://www.arb-silva.de/), and read alignments were filtered to retain those expanding beyond 80% of the read length (≥100 nt) with ≥99% sequence identity. Differential abundance in phylum, family, and genus distribution was evaluated with Linear Mixed Models (LMM) by using time points as fixed effects and subject-specific information (gender, age, and BMI) as random effects. Additionally, we assessed the taxonomy distribution of the coding metagenes with differential abundance by using BLAST and the NCBI nonredundant protein database (ftp://ftp.ncbi.nlm.nih.gov/refseq/). Taxonomy identification for those metagenes was based on selection of hits with the best alignment score among the multiple alignments (covering 100% query sequence and at least 70% sequence identity) of each query against targets retrieved. Cases were defined as “uncertain” when equal scoring was obtained in comparison with more than one hit belonging to different microbial species.

**(iii) Functional analyses.** Preliminary analysis to assess the function of differentially abundant metagenes was completed by submitting the amino acid sequence of metagenes with significant increasing and decreasing abundance, as a consequence of the AXOS intake, to the Kyoto Encyclopedia of Genes and Genomes (KEGG) Automatic Annotation Server (KAAS) (63). Advanced functional enrichment analysis was performed by annotating the full set of nonredundant coding sequences, obtained in the global metagenome assembly, against the Pfam database (64) through the WebMGA server (65). Functional enrichment of Pfam functions in samples after AXOS intake was evaluated by hypergeometric Fisher's exact test with correction for multiple testing using the Benjamini-Hochberg method.

**(iv) Biochemical and lipidomic data.** Plasma biochemical markers previously assessed (21) were reanalyzed in the present study for the selected subset of subjects. The Shapiro-Wilk normality test was estimated for all variables, and accordingly, paired and one-sided *t* test or Wilcox signed-rank test was performed, respectively, in order to establish if the AXOS intervention improved any of the biochemical parameters measured. To establish possible relationships between changes in the abundance of bacterial genera and markers of glucose metabolism (HOMA-IR and fasting insulin), a logistic regression model was applied, using the *glm* [family=“binomial”(link=“logit”)] function of R v3.4.3; the HOMA-IR index was used as binary outcome (1 = improved and 0 = nonimproved), and the changes in the relative abundance of different bacterial genera (Δ_genus_ = log_10_ normalized reads at endpoint − log_10_ normalized reads at start point) were used as explanatory variable. Lipidomics data were analyzed using Wilcox signed-rank test for paired samples with Benjamini-Hochberg correction for multiple comparisons when appropriate.

**(v) Human virome analysis.** Raw fastq sequences were processed using the ViromeScan software to taxonomically characterize the virome directly from metagenomic reads (22) using the *-d human_DNA* parameter to detect only DNA viruses with humans as a natural host. The relative abundance was used to perform statistical analyses and comparisons among samples before and after the AXOS intervention. Alpha-diversity was computed for each sample considering the number of viral species detected within each metagenome. Significance testing was performed using the R package stats and nonparametric Wilcox signed-rank test for paired samples. When appropriate, *P* values were adjusted for multiple comparisons using the Benjamini-Hochberg correction. A false-discovery rate (FDR) of <0.05 was considered statistically significant. All the statistics and plots for metagenomics and functional approaches were obtained and designed on R v3.4.3.

### Plasma and fecal lipidomics. (i) Quantification of plasma lipid species.

Lipids were quantified by direct flow injection electrospray ionization tandem mass spectrometry (ESI-MS/MS) in positive ion mode using the analytical setup and strategy described previously (66, 67). Lipid extraction was performed according to the method of Bligh and Dyer (68), in the presence of non-naturally occurring lipid species as internal standards. The following lipid species were added as internal standards: phosphatidylcholine (PC) 14:0/14:0, PC 22:0/22:0, phosphatidylethanolamine (PE) 14:0/14:0, PE 20:0/20:0 (diphytanoyl), phosphatidylinositol (PI) 17:0/17:0, lysophosphatidylcholine (LPC) 13:0, LPC 19:0, sphingosine-based ceramides (Cer) d18:1/14:0, Cer 17:0, D7-free cholesterol (FC), cholesteryl ester (CE) 17:0, and CE 22:0. A fragment ion of *m/z* 184 was used for PC, sphingomyelin (SM) (67), and LPC (69). Neutral loss fragments were used for the following lipid classes: PE and PI with a loss of 141 and 277, respectively (70, 71). PE-based plasmalogens (PE P) were analyzed according to the principles described by Zemski Berry and Murphy (72). Cer and hexosylceramides (HexCer) were analyzed using a fragment ion of *m/z* 264 (73). Free cholesterol (FC) and CE were quantified using a fragment ion of *m/z* 369 after selective derivatization of FC (66). Quantification was achieved using two non-naturally occurring internal standards (IS) for each lipid class (except for PI, sphingomyelin [SM] was calculated using PC IS and PE P were calculated using PE IS), and calibration lines were generated by standard addition of a number of naturally occurring species to plasma. Calibration lines were generated for the following naturally occurring species: PC 34:1, 36:2, 38:4, and 40:0 and PC O-16:0/20:4; SM d18:1/16:0, 18:1, and 18:0; LPC 16:0, 18:1, and 18:0; PE 34:1, 36:2, 38:4, and 40:6 and PE P 16:0/20:4; Cer d18:1/16:0, 18:0, 20:0, 24:1, and 24:0; FC, CE 16:0, 18:2, 18:1, and 18:0. Deisotoping and data analysis for all lipid classes were performed by self-programmed Excel macros as described previously (67, 74). Lipid species were annotated according to the recently published proposal for shorthand notation of lipid structures that are derived from mass spectrometry (75). Glycerophospholipid species annotation was based on the assumption of even-numbered carbon chains only. SM species annotation is based on the assumption that a sphingoid base with two hydroxyl groups is present.

**(ii) Quantification of bile acids.** Both plasma and fecal bile acids were quantified by LC-MS/MS using stable isotope dilution analysis. Fecal samples were homogenized as described below using 10-fold dilution of the samples.

**(iii) Quantification of fecal lipid species.** Fecal samples were homogenized in 70% 2-propanol, using a gentleMACS dissociator (Miltenyi Biotec GmbH, Bergisch Gladbach, Germany) as described previously (76). Fecal sterols and 5α/β-stanols were quantified by liquid chromatography–high-resolution mass spectrometry (LC-MS/HRMS) after derivatization to *N*,*N*-dimethylglycine esters (76). Fecal fatty acids were quantified by gas chromatography coupled to mass spectrometry (GC-MS) after preparation of fatty acid methyl esters (77).

### Metabolomics. (i) Sample preparation.

In order to reduce the water signal, urine samples (1 ml) were freeze-dried and reconstituted in 650 μl NMR phosphate buffer [sodium phosphate, 0.2 M, pH 7.4; sodium 3-(trimethylsilyl)-propionate-2,2,3,3-d4 [TSP] [Sigma-Aldrich], 1 mM; 80% D_2_O, 20% H_2_O). TSP served as NMR reference. Feces (200 mg) were homogenized in 800 μl of NMR buffer for 5 min at 25 Hz in a tissue lyser (Qiagen). Plasma samples (350 μl) were mixed with D_2_O (350 μl). All homogenized samples were centrifuged (10 min, 4°C, 16,000 × *g*) and transferred to 5-mm NMR tubes (Bruker, United Kingdom) for analysis by NMR spectroscopy.

**(ii) Spectrum acquisition.** The NMR experiments were carried out in the Chemical Analysis Facility (CAF; University of Reading) using a Bruker AV700 NMR instrument equipped with a 5-mm inverse CryoProbe, for increased sensitivity. A standard one-dimensional nuclear Overhauser effect spectroscopy (NOESY-PR-1D) experiment was performed on all three types of samples, using a standard preset pulse sequence (noesy1d90°). Additionally, a Carr-Purcell-Meiboom-Gill (CPMG) experiment was applied to plasma samples, where simple presaturation of the water peak was used. This experiment reduced the signal contribution from albumin and lipoproteins present in plasma and highlighted signals from smaller molecules. All samples were analyzed at 300 K, and a 65,000-data-point spectrum (spectral width, 14,705 Hz) was obtained by recording 128 scans (8 dummy scans).

**(iii) Data processing and statistical analysis.** Phase and baseline of the spectra were corrected using MestreNova software (version 10.0m; MestreLab Research). NMR spectra were referenced to TSP peak for urine and fecal water samples and to glucose (at δ 5.223 ppm) for plasma samples. The processed spectra were digitalized and transferred to Matlab (version R2017a; MathWorks, Natick, MA) for the statistical analysis. The residual water signal was removed, and all spectra were normalized to the total spectral area for feces and urine. Plasma spectra were not normalized. Relative spectra were mean centered and scaled to unit variance. For the first stage of the analysis (unsupervised), principal-component analysis (PCA) was used. The next, supervised stage of the analysis involved the orthogonal projection to latent structure discriminant analyses (O-PLS-DA) (no orthogonal components used) to compare the changes in metabolite profiles between the two time points. The comparison was made between the baseline (start point) and the endpoint of AXOS intervention.

NMR spectra were used as a matrix of variables *X* and time point vector *Y* (0, baseline; 1, endpoint) as a predictor. This analysis was used to construct a model identifying metabolites differentiating between the two time points. The internal validation of the model was evaluated using the following parameters: the goodness of fit (*R*^2^*Y*), showing what percentage of variation is explained by the model, and goodness of prediction (*Q*^2^*Y*), the percentage of *Y* predicted after 7-cross validation. For the correlation of metabolome with microbiome data, the metagenomics readings (normalized using the “reads per million” approach) were used as a predictor *Y* using the O-PLS analysis.

### Data availability.

The raw fastq sequences generated from the shotgun sequencing of fecal DNA are publicly available at the ENA under the project accession number PRJEB25727.
