# Supplementary figures and images for: A Multi-omics Approach to Unraveling the Microbiome-Mediated Effects of Arabinoxylan Oligosaccharides in Overweight Humans
Source: mSystems. 2019 May 28;4(4):e00209-19. doi: 10.1128/mSystems.00209-19 (PMC6538848; doi:10.1128/mSystems.00209-19)

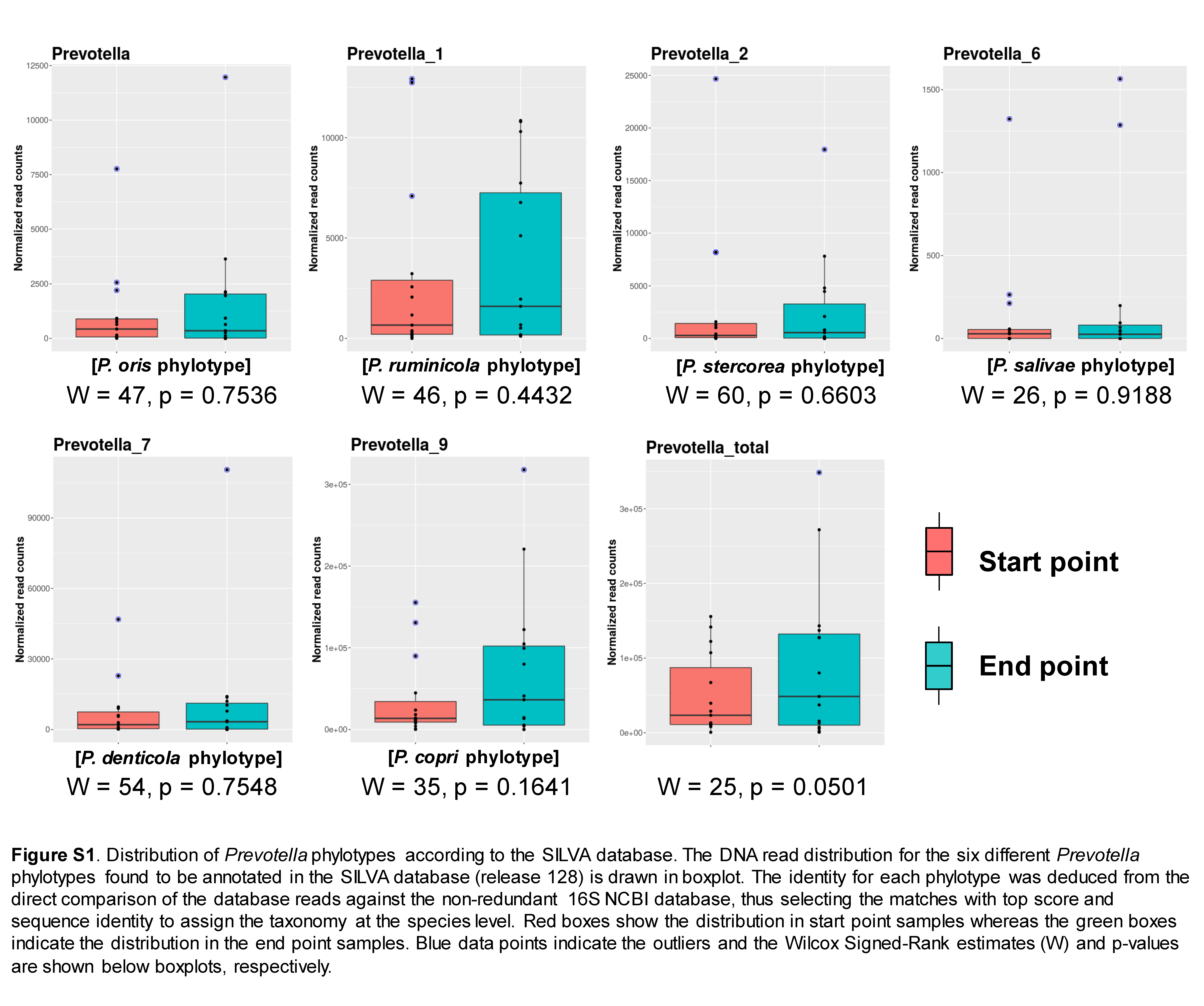

Supplement: FIG S1 [file mSystems.00209-19-sf001.tif]

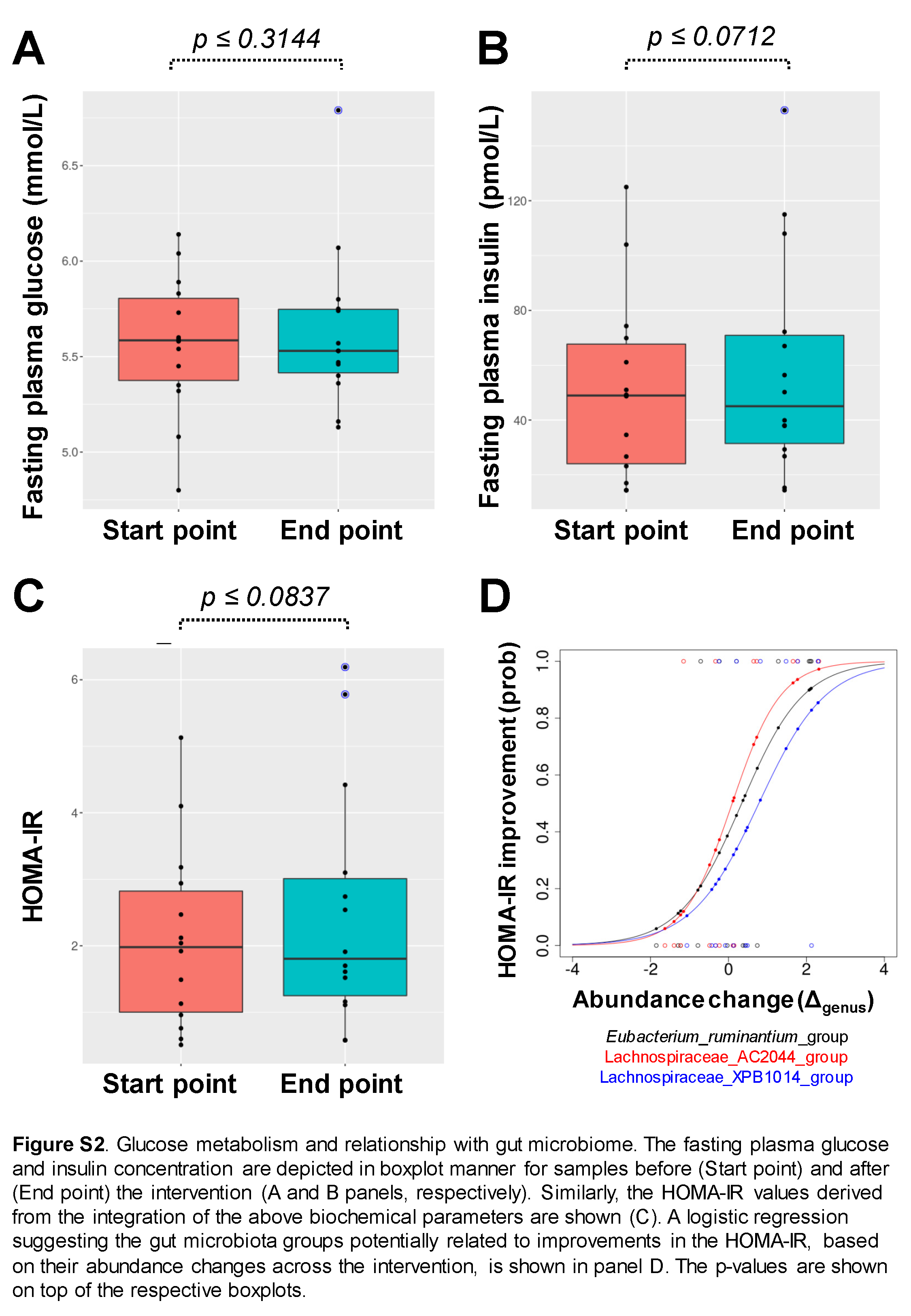

Supplement: FIG S2 [file mSystems.00209-19-sf002.tif]
