# Supplementary material for: A Multi-omics Approach to Unraveling the Microbiome-Mediated Effects of Arabinoxylan Oligosaccharides in Overweight Humans
Source: mSystems. 2019 May 28;4(4):e00209-19. doi: 10.1128/mSystems.00209-19 (PMC6538848; doi:10.1128/mSystems.00209-19)
Supplement: TABLE S1 [file mSystems.00209-19-st001.docx]

Table S1. Characteristics for the participants involved in the multi-omics assessment (N=15)^1^

|  | **Median** | **Q1 ; Q3** |
| --- | --- | --- |
| Age | 50 | 36 ; 52.5 |
| Women (%) | 80 | - |
| Anthropometric |  |  |
| Body weight (kg) | 86.7 | 81.8 ; 91.4 |
| BMI (kg/m^2^) | 29.3 | 27.9 ; 31.2 |
| FM (kg) | 26.1 | 18.7 ; 33.2 |
| LBM (kg) | 56.9 | 51.4 ; 68.7 |
| Fat percent, whole body (%) | 30.0 | 21.5 ; 38.3 |
| Waist circumference (cm) | 94.8 | 92.1 ; 100.8 |
| Hip circumference (cm) | 112.5 | 109.1 ; 114.9 |
| Sagittal height (cm) | 22.4 | 20.6 ; 23.8 |
| Blood pressure |  |  |
| Systolic (mmHg) | 118.5 | 105.5 ; 126.0 |
| Diastolic (mmHg) | 78.0 | 75.5 ; 83.5 |
| Pulse (beats/min) | 62.0 | 55.3 ; 66.0 |
| Lipid profile^2^ |  |  |
| Total CHO (mmol/L) | 5.17 | 4.25 ; 5.48 |
| HDL-CHO (mmol/L) | 1.48 | 1.32 ; 1.61 |
| LDL-CHO (mmol/L) | 3.06 | 2.10 ; 3.36 |
| VLDL-CHO (mmol/L) | 0.56 | 0.41 ; 0.72 |
| TG (mmol/L) | 1.12 | 0.87 ; 1.34 |
| Glucose metabolism^2^ |  |  |
| Glucose (mmol/L) | 5.59 | 5.38 ; 5.81 |
| Insulin (pmol/L) | 48.9 | 24.1 ; 67.7 |
| HOMA-IR | 1.98 | 1.00 ; 2.82 |
| HOMA-β | 73.0 | 37.7 ; 106.8 |
| Inflammatory markers^2^ |  |  |
| hsCRP (mg/L) | 1.66 | 1.05 ; 2.27 |
| Hb (mmol/L) | 8.40 | 7.80 ; 9.05 |
| WBC (10^9^/L) | 5.60 | 4.33 ; 6.78 |
| Liver markers^2^ |  |  |
| ASAT (U/L) | 21.0 | 19.0 ; 24.3 |
| ALAT (U/L) | 21.0 | 14.8 ; 26.5 |

1Data are given for baseline (Start point) as median with Q1 ; Q3 distribution.

2 N=14, enough blood sample from one participant could not be obtained for biochemical analysis by technical reasons.

ALAT, alanine aminotransferase; ASAT, aspartate aminotransferase; BMI, body mass index; CHO, cholesterol; FM, fat mass; Hb, hemoglobin; HDL, high density lipoprotein; HOMA-β, homeostatic model assessment - beta-cell function; HOMA-IR, homeostatic model assessment- insulin resistance; hsCRP, high sensitive C-reactive protein; LBM, lean body mass; LDL, low density lipoprotein; TG, triglycerides; VLDL, very low density lipoprotein; WBC, white blood cell count.
