# Supplementary material for: A Multi-omics Approach to Unraveling the Microbiome-Mediated Effects of Arabinoxylan Oligosaccharides in Overweight Humans
Source: mSystems. 2019 May 28;4(4):e00209-19. doi: 10.1128/mSystems.00209-19 (PMC6538848; doi:10.1128/mSystems.00209-19)
Supplement: TABLE S2 [file mSystems.00209-19-st002.docx]

Table S2. KEGG metabolic pathways associated with the down-represented metagenes

| **KEEG pathway (ko number and description)** | **Number of metagenes annotated** |
| --- | --- |
| ko01100 Metabolic pathways | 71 |
| ko01110 Biosynthesis of secondary metabolites | 25 |
| ko01120 Microbial metabolism in diverse environments | 25 |
| ko02010 ABC transporters | 20 |
| ko01130 Biosynthesis of antibiotics | 20 |
| ko02020 Two-component system | 17 |
| ko02024 Quorum sensing | 16 |
| ko00230 Purine metabolism | 15 |
| ko01230 Biosynthesis of amino acids | 15 |
| ko01200 Carbon metabolism | 14 |
| ko00970 Aminoacyl-tRNA biosynthesis | 13 |
| ko00520 Amino sugar and nucleotide sugar metabolism | 11 |
| ko03440 Homologous recombination | 10 |
| ko00500 Starch and sucrose metabolism | 9 |
| ko00620 Pyruvate metabolism | 9 |
| ko00250 Alanine, aspartate and glutamate metabolism | 9 |
| ko03430 Mismatch repair | 8 |
| ko00720 Carbon fixation pathways in prokaryotes | 8 |
| ko03030 DNA replication | 8 |
| ko00240 Pyrimidine metabolism | 7 |
| ko00680 Methane metabolism | 7 |
| ko00550 Peptidoglycan biosynthesis | 7 |
| ko00051 Fructose and mannose metabolism | 6 |
| ko04112 Cell cycle - Caulobacter | 6 |
| ko00030 Pentose phosphate pathway | 6 |
| ko03018 RNA degradation | 5 |
| ko00650 Butanoate metabolism | 5 |
| ko03420 Nucleotide excision repair | 5 |
| ko03410 Base excision repair | 5 |
| ko00010 Glycolysis / Gluconeogenesis | 5 |
| ko02026 Biofilm formation - Escherichia coli | 5 |
| ko01210 2-Oxocarboxylic acid metabolism | 4 |
| ko00710 Carbon fixation in photosynthetic organisms | 4 |
| ko00910 Nitrogen metabolism | 4 |
| ko00020 Citrate cycle (TCA cycle) | 4 |
| ko00511 Other glycan degradation | 4 |
| ko00220 Arginine biosynthesis | 4 |
| ko00640 Propanoate metabolism | 4 |
| ko01501 beta-Lactam resistance | 4 |
| ko00052 Galactose metabolism | 3 |
| ko03070 Bacterial secretion system | 3 |
| ko02025 Biofilm formation - Pseudomonas aeruginosa | 3 |
| ko00630 Glyoxylate and dicarboxylate metabolism | 3 |
| ko00270 Cysteine and methionine metabolism | 3 |
| ko00460 Cyanoamino acid metabolism | 3 |
| ko00450 Selenocompound metabolism | 3 |
| ko00260 Glycine, serine and threonine metabolism | 3 |
| ko03010 Ribosome | 3 |
| ko00760 Nicotinate and nicotinamide metabolism | 3 |
| ko00300 Lysine biosynthesis | 3 |
| ko00670 One carbon pool by folate | 2 |
| ko05111 Biofilm formation - Vibrio cholerae | 2 |
| ko00860 Porphyrin and chlorophyll metabolism | 2 |
| ko00430 Taurine and hypotaurine metabolism | 2 |
| ko00513 Various types of N-glycan biosynthesis | 1 |
| ko00730 Thiamine metabolism | 1 |
| ko00770 Pantothenate and CoA biosynthesis | 1 |
| ko02040 Flagellar assembly | 1 |
| ko00290 Valine, leucine and isoleucine biosynthesis | 1 |
| ko00940 Phenylpropanoid biosynthesis | 1 |
| ko04724 Glutamatergic synapse | 1 |
| ko00600 Sphingolipid metabolism | 1 |
| ko00750 Vitamin B6 metabolism | 1 |
| ko00471 D-Glutamine and D-glutamate metabolism | 1 |
| ko01503 Cationic antimicrobial peptide (CAMP resistance) | 1 |
| ko00564 Glycerophospholipid metabolism | 1 |
| ko00261 Monobactam biosynthesis | 1 |
| ko04979 Cholesterol metabolism | 1 |
| ko01051 Biosynthesis of ansamycins | 1 |
| ko02030 Bacterial chemotaxis | 1 |
| ko01523 Antifolate resistance | 1 |
| ko00920 Sulfur metabolism | 1 |
| ko00531 Glycosaminoglycan degradation | 1 |
| ko00480 Glutathione metabolism | 1 |
| ko00791 Atrazine degradation | 1 |
| ko00540 Lipopolysaccharide biosynthesis | 1 |
