# Supplementary material for: A Multi-omics Approach to Unraveling the Microbiome-Mediated Effects of Arabinoxylan Oligosaccharides in Overweight Humans
Source: mSystems. 2019 May 28;4(4):e00209-19. doi: 10.1128/mSystems.00209-19 (PMC6538848; doi:10.1128/mSystems.00209-19)
Supplement: TABLE S3 [file mSystems.00209-19-st003.docx]

Table S3. Functional enrichment analysis in the over-represented metagenomes.

| Domain^1^ | Annotation^1^ | OR^2^ | FDR^3^ |
| --- | --- | --- | --- |
| PF12875 | DUF3826, putative sugar binding domain | 98.8 | 0.0456 |
| PF12128 | DUF3584, AAA-type ATPase | 69.7 | 0.0049 |
| PF00686 | CBM_20, starch binding domain | 43.9 | 0.0001 |
| PF01637 | ATPase_2, ATPase domain | 18.4 | 2.07^-6^ |
| PF03175 | DNA_pol_B_2, DNA polymerase type B | 16.4 | 0.0268 |
| PF15495 | Fimbrillin_C, Major fimbrial subunit protein | 16.4 | 0.0268 |
| PF13734 | Inhibitor_I69, Spi protease inhibitor | 15.3 | 0.0331 |
| PF16738 | CBM26, Starch binding module 26 | 14.9 | 0.0342 |
| PF12008 | EcoR124_C, Type I restriction and modification enzyme | 13.4 | 0.0456 |
| PF03422 | CBM_6, Carbohydrate binding module (family 6) | 13.0 | 0.0035 |
| PF08011 | PDDEXK_9, PD-(D/E)XK nuclease superfamily | 12.1 | 4.13^-8^ |
| PF14905 | OMP_b_brl_3, Outer membrane protein beta-barrel family | 11.3 | 2.51^-8^ |
| PF07676 | PD40, WD40-like Beta Propeller Repeat | 10.9 | 0.0021 |
| PF07495 | Y_Y_Y, domain of unsaturated carbohydrate periplasmic sensor | 10.0 | 3.12^-7^ |
| PF16353 | DUF4981, Beta-galactosidase activity associated domain | 9.7 | 0.0125 |
| PF05272 | VirE, Virulence-associated protein E like protein | 9.6 | 0.0391 |
| PF04313 | HSDR_N, Type I restriction enzyme R protein | 8.8 | 0.0058 |
| PF00593 | TonB_dep_Rec, TonB dependent receptor | 8.5 | 1.93^-30^ |
| PF09820 | AAA-ATPase_like, Predicted AAA-ATPase | 8.3 | 7.43^-7^ |
| PF16355 | DUF4982, Beta-galactosidase activity associated domain | 8.2 | 0.0261 |
| PF13715 | CarboxypepD_reg_2, Carboxypeptidase regulatory-like domain | 7.8 | 1.93^-30^ |
| PF02929 | Bgal_small_N, Beta galactosidase small chain | 7.7 | 0.0044 |
| PF07715 | Plug, TonB-dependent receptor | 7.6 | 7.46^-30^ |
| PF13173 | AAA_14, AAA ATPase domain | 7.1 | 9.34^-8^ |
| PF13620 | CarboxypepD_reg, Carboxypeptidase regulatory-like domain | 6.8 | 1.64^-20^ |
| PF00150 | Cellulase, Glycosyl hydrolase family 5 | 6.7 | 0.0039 |
| PF12771 | SusD-like_2, Starch-binding associating with outer membrane | 6.5 | 0.0272 |
| PF02837 | Glyco_hydro_2_N, Glycosyl hydrolase family 2 | 6.2 | 5.77^-7^ |
| PF07944 | Glyco_hydro_127, Beta-L-arabinofuranosidase GH127 | 6.2 | 0.0336 |
| PF13635 | DUF4143, ATPase activity associated domain | 5.7 | 0.0049 |
| PF00703 | Glyco_hydro_2, Glycosyl hydrolase family 2 | 5.5 | 2.19^-5^ |
| PF02836 | Glyco_hydro_2_C, Glycosyl hydrolase family 2 C-ter | 5.2 | 0.0001 |
| PF04616 | Glyco_hydro_43, Glycosyl hydrolase family 43 | 5.2 | 0.0011 |
| PF14322 | SusD-like_3, Starch-binding associating with outer membrane | 4.8 | 6.86^-9^ |
| PF01915 | Glyco_hydro_3_C, Glycosyl hydrolase family 3 C-ter | 4.8 | 0.0043 |
| PF14310 | Fn3-like, Fibronectin type III-like domain | 4.6 | 0.0110 |
| PF07980 | SusD_RagB, SusD family | 4.5 | 8.57^-8^ |
| PF01580 | FtsK_SpoIIIE, cell division protein | 4.5 | 0.0433 |
| PF07494 | Reg_prop, Two component regulator propeller | 4.5 | 0.0439 |
| PF02321 | OEP, Outer membrane efflux protein | 4.3 | 0.0017 |
| PF00933 | Glyco_hydro_3_N, Glycosyl hydrolase family 3 N-ter | 3.9 | 0.0220 |
| PF09479 | Flg_new, Listeria-Bacteroides repeat domain | 2.9 | 0.0003 |
| PF04851 | ResIII, Type III restriction enzyme | 2.8 | 0.0252 |
| PF01381 | HTH_3, Helix-turn-helix transcriptional regulator | 0.2 | 0.0023 |
| PF12844 | HTH_19, Helix-turn-helix transcriptional regulator | 0.1 | 0.0035 |
| PF13560 | HTH_31, Helix-turn-helix transcriptional regulator | 0.1 | 0.0132 |
| PF13443 | HTH_26, Cro/C1-type HTH DNA-binding domain | 0.1 | 0.0342 |
| PF00395 | SLH, S-layer homology domain - glycoproteins of bacterial surface | 0.0 | 0.0146 |

1 Domain identifier and functional annotation according to information retrieved from Pfam database (http://pfam.xfam.org/).

2 Odds ratio (OR) calculated from the Fisher's exact test under conditional Maximum Likelihood Estimate (MLE) approach.

3 False discovery rate (FDR) obtained after multiple testing correction of all domains detected (N = 7,711 domains)
