# Supplementary material for: A Multi-omics Approach to Unraveling the Microbiome-Mediated Effects of Arabinoxylan Oligosaccharides in Overweight Humans
Source: mSystems. 2019 May 28;4(4):e00209-19. doi: 10.1128/mSystems.00209-19 (PMC6538848; doi:10.1128/mSystems.00209-19)
Supplement: TABLE S4 [file mSystems.00209-19-st004.docx]

Table S4. Changes in lipid profiles from plasma samples.

| Lipid species | Lipid subspecies | Start point  (μmol/L median) | End point  (μmol/L median) | Delta values | p-value^1^ |
| --- | --- | --- | --- | --- | --- |
| Cholesteryl esters | CE 20:0 | 1.088 | 1.348 | 0.260 | 0.0014 |
| Ceramides | HexCer d18:1/16:0 | 0.733 | 0.570 | -0.163 | 0.0011 |
|  | HexCer d18:1/24:1 | 1.077 | 0.836 | -0.263 | 0.0011 |
|  | Cer d18:1/23:0 | 0.8835 | 0.794 | -0.09 | 0.0120 |
|  | Cer d18:1/24:0 | 3.031 | 2.899 | -0.132 | 0.0901 |
|  | Cer d18:1/24:1 | 1.516 | 1.457 | -0.059 | 0.1673 |
|  | Cer d18:1/20:0 | 0.173 | 0.18 | 0.007 | 0.4897 |
|  | Cer d18:1/22:0 | 0.988 | 0.975 | -0.014 | 0.4899 |
|  | Cer d18:1/18:0 | 0.205 | 0.196 | -0.009 | 0.576 |
|  | Cer d18:1/16:0 | 0.507 | 0.503 | -0.004 | 0.8017 |
|  | **Total Cer** | **7.504** | **7.423** | **-0.081** | **0.0788** |

1 p-values are based on comparison using Wilcox Signed-Rank test and corrected by multiple testing using the Benjamini-Hochberg method.

Cer = Ceramide, HexCer = hexosylceramide, CE = Cholesteryl ester.
